# Hedge Scope Detection in Biomedical Texts: An Effective Dependency-Based Method

**DOI:** 10.1371/journal.pone.0133715

**Published:** 2015-07-28

**Authors:** Huiwei Zhou, Huijie Deng, Degen Huang, Minling Zhu

**Affiliations:** 1 School of Computer Science and Technology, Dalian University of Technology, Dalian, Liaoning, China; 2 School of Computer, Beijing Information Science and Technology University, Beijing, China; University Hospitals of Geneva, SWITZERLAND

## Abstract

Hedge detection is used to distinguish uncertain information from facts, which is of essential importance in biomedical information extraction. The task of hedge detection is often divided into two subtasks: detecting uncertain cues and their linguistic scope. Hedge scope is a sequence of tokens including the hedge cue in a sentence. Previous hedge scope detection methods usually take all tokens in a sentence as candidate boundaries, which inevitably generate a large number of negatives for classifiers. The imbalanced instances seriously mislead classifiers and result in lower performance. This paper proposes a **dependency-based candidate boundary selection method** (DCBS), which selects the most likely tokens as candidate boundaries and removes the exceptional tokens which have less potential to improve the performance based on dependency tree. In addition, we employ the composite kernel to integrate lexical and syntactic information and demonstrate the effectiveness of structured syntactic features for hedge scope detection. Experiments on the CoNLL-2010 Shared Task corpus show that our method achieves 71.92% F1-score on the golden standard cues, which is 4.11% higher than the system without using DCBS. Although the candidate boundary selection method is only evaluated on hedge scope detection here, it can be popularized to other kinds of scope learning tasks.

## Introduction

Hedges are linguistic devices that indicate uncertain or unreliable information. Hedged information is usually used in science texts, especially in the biomedical domain to express impressions or hypothesized explanations of experimental results. According to the statistics on BioScope corpus [1], 17.69% of the sentences in abstract section and 22.29% of the sentences in full paper section contain speculative fragments. In order to distinguish factual and uncertain information, detecting hedged information is an increasingly important task in biomedical information extraction. The CoNLL-2010 Shared Task [2] is dedicated to the detection of uncertain information. The shared task contains two subtasks: Task 1 aims to identify hedge cues and Task 2 devotes to detecting the in-sentence scope of a given cue.

A hedged sentence taken from the CoNLL-2010 Shared Task corpus is shown as follows:

Sentence 1: *Our data indicate that < xscope > the mutagenic DNA deaminases are < cue > potentially < /cue > an important target for hormonal regulation < /xscope >*.

The token “*potentially*”, namely hedge cue, indicates that the following statement is not backed up by facts. The in-sentence scope of the hedge cue “*potentially*” is the statement “*the mutagenic DNA deaminases are potentially an important target for hormonal regulation*”.

Researches on hedge cue identification have been developed rapidly [3–5]. However, the results of hedge scope detection are not satisfied. Hedge scope detection is a difficult task, since it falls within the scope of semantic analysis of sentences exploiting syntactic patterns. This paper focuses on the hedge scope detection task.

Generally, hedge scope detection approaches can be divided into two categories: rule based methods and machine learning based methods. Rule based methods detect scope by constructing syntactic rules. Özgür et al. [6] and Øvrelid et al. [7] manually compiled rules, and Apostolova et al. [8] and Zhou et al. [9] automatically extracted rules based on syntactic structures. Rule based methods could make full use of syntactic information and have achieved good performance in the specific resource, but the extracted rules are hard to be developed to a new resource.

Machine learning based methods formulate the hedge scope detection task as a token classification problem, which usually classify each token in a sentence as being the first element of the scope (F-scope), the last (L-scope), or neither (None). We refer to such traditional token-based methods as TTB in this article. Morante and Daelemans [10] utilized lexical and contextual features to learn three classifiers to predict F-scope, L-scope, and None respectively. Morante et al. [11] extended this work by extracting flat dependency features to improve the detection performance. They achieved the highest F1-score (57.32%) on CoNLL-2010 Shared Task 2. Velldal et al. [12] combined manual rules and machine learning to exploit syntactic and surface-oriented information for the scope detection task.

TTB regards scope boundary tokens as positives, and the others as negatives for scope boundary classifiers. For example, the F-scope classifier takes the F-scope tokens as positives and the others on the left side of a given cue (including the cue itself) as negatives. This inevitably generates plenty of negatives. Excessive negatives mislead classifier and degrade classification performance. Zhu et al. [13] altered the classification unit from token to phrase by formulating the scope learning as shallow semantic parsing problem, which decreased candidate instances significantly. However, regarding the great granularity phrase as candidate unit is too coarse or general to capture semantic information.

For both rule based and machine learning based methods, syntactic information plays an important role in hedge scope detection. Tree kernel [14] can capture structured syntactic information and has been applied in various NLP tasks like relation extraction [15, 16], semantic role labeling [17], events extraction [18] and drug-drug interaction detection [19, 20], etc. Zhou et al. [21] applied tree kernel with structured phrase features to hedge scope resolution task. Zou et al. [22] utilized tree kernel to capture phrase and dependency structure information to optimize the scope detection performance. However, adjacent tokens in a sentence are hard to be classified by tree kernel, as adjacent tokens have similar structure and contextual information.

This paper proposes a dependency-based candidate boundary selection method (DCBS) to decrease candidate instances and enhance the discriminability of instances for classifiers effectively. DCBS selects the most likely tokens as candidate boundary and removes the exceptional tokens which have less potential to improve the performance based on dependency tree. Furthermore, the composite kernel consisting of the polynomial kernel and the tree kernel is employed to capture lexical and syntactic structure information. Although our approach is only evaluated on hedge scope detection task in this paper, it is portable to other kinds of scope learning tasks.

## Materials and Methods

**Fig 1:**
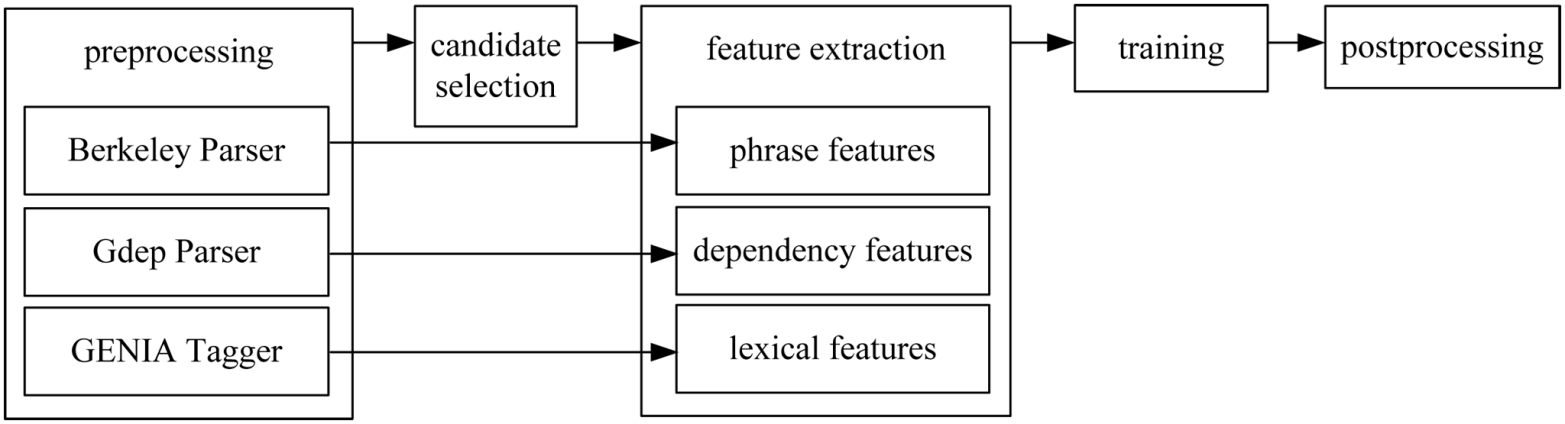
System architecture.

Our scope detection system consists of five steps: preprocessing, candidate selection, feature extraction, training and postprocessing as shown in Fig 1. In the preprocessing step, the CoNLL-2010 Shared Task corpus are processed with Berkeley Parser (http://code.google.com/p/berkeyparser/), Gdep Parser (http://pepple.ict.usc.edu/ sagae/parser/gdep/) and GENIA Tagger (http://www-tsujii.is.s.utokyo.ac.jp/GENIA/tagger/) to get phrase tree, dependency tree and lexical information, respectively. In the candidate selection step, we use DCBS to select candidate boundaries. In the feature extraction step, phrase features, dependency features and lexical features are extracted based on phrase tree, dependency tree and lexical information, which are generated in the preprocessing step. In the training step, classifiers are learned by using SVM-LIGHT-TK 1.2 toolkit (http://disi.unitn.it/moschitti/Tree-Kernel.htm) that provides the convolution tree kernel. Finally, the postprocessing rules are adopted to get the complete sequence of the scope in the postprocessing step. In the following part of this section, we will describe the detailed implementation of the hedge scope detection system.

### Dependency-based Candidate Boundary Selection Method

**Fig 2:**
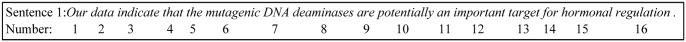
The sequence number of tokens in sentence 1.

**Fig 3:**
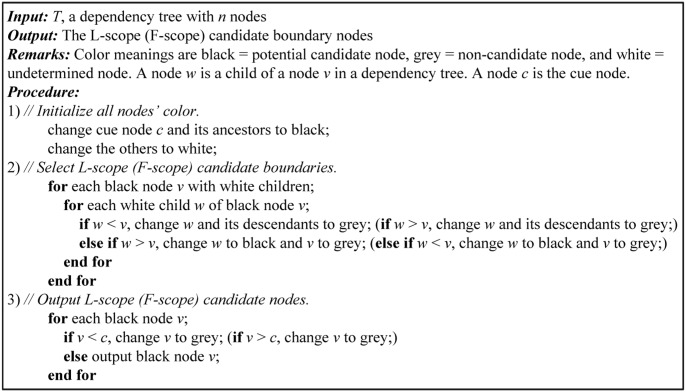
The L-scope (F-scope) candidate boundary selection algorithm.

Token dependency reflects semantic modification relationships of in-sentence tokens. For two tokens with dependency relation, if one token falls within the hedge scope, the other is likely to be in the scope too. Therefore, the left one of the two tokens with dependency relation is more probable as the F-scope candidate boundary than the right one. The right one is not necessary to be selected as the F-scope candidate boundary and should be eliminated from F-scope candidates. Similarly, the right one of the two tokens with dependency relation is more likely as the L-scope candidate, and the left one should be eliminated from L-scope candidates. According to these analyses, we propose a dependency-based candidate boundary selection method (DCBS) for hedge scope detection. To concisely describe DCBS, each token in a sentence is numbered sequentially as shown in Fig 2. The L-scope (F-scope) candidate boundary selection algorithm based on dependency tree is described in Fig 3.

**Fig 4:**
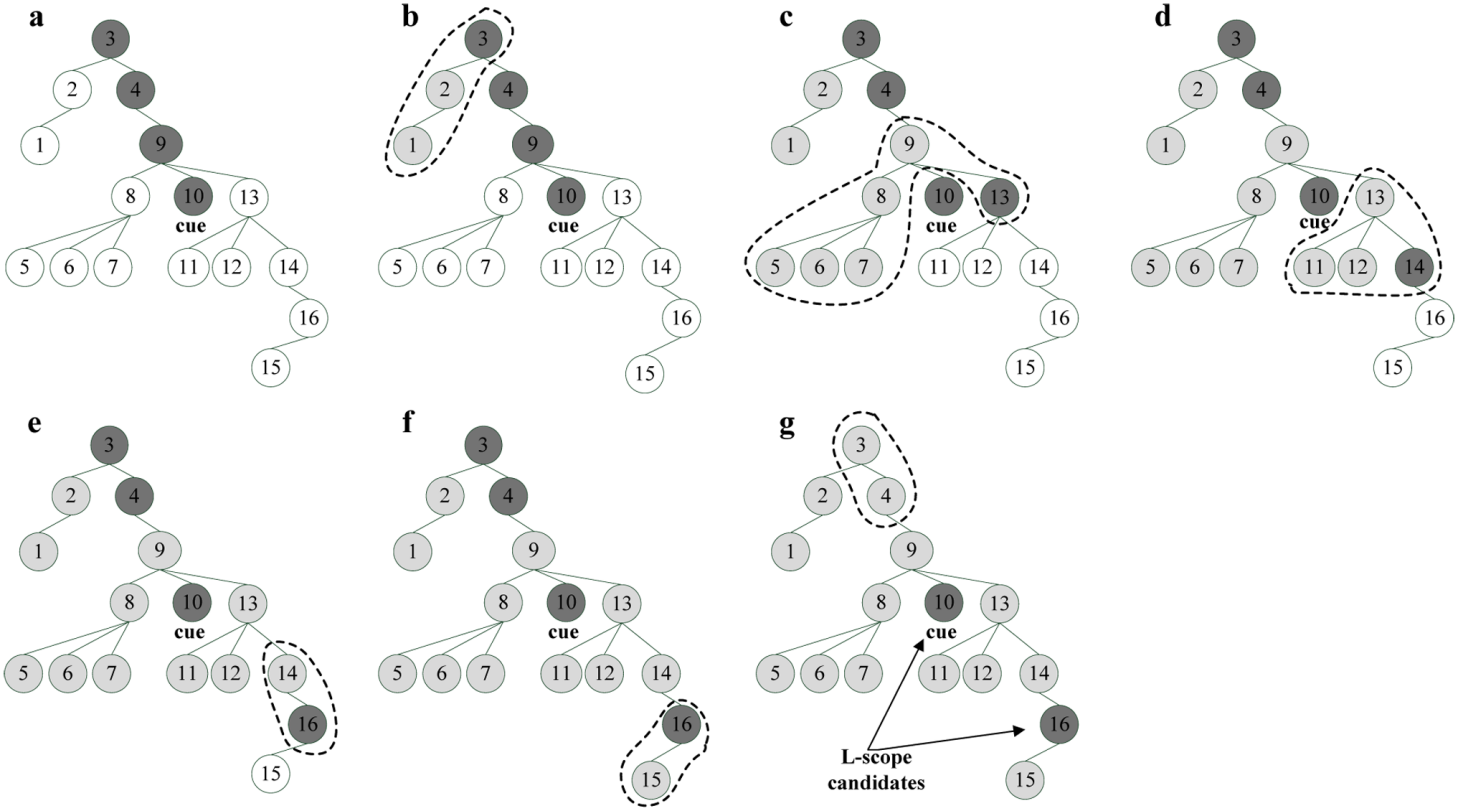
An example of the L-scope candidate selection process with DCBS. (a) Initialize all nodes’ color; Select the L-scope candidate boundary from (b) to (f); (g) Output the L-scope candidate nodes.

Taking the sentence 1 as an example, the L-scope candidate selection process with DCBS is shown in Fig 4. A brief description is as follows:

1. Initial all nodes’ color as shown in Fig 4a. Change cue node 10 and its ancestors (node 3, 4, 9) to black. Change the others to white.
2. Select the L-scope candidate boundaries from Fig 4b to 4f one by one. For each black node with white children, the black node is compared to each of its white children. If its white child is smaller than the black node, change its white child and the descendants of its white child to grey. If its white child is larger than the black node, change its white child to black and the black node to grey. The changes of node’s color are shown in the dash line in Fig 4 (Fig 4b for node 3, Fig 4c for node 9, Fig 4d for node 13, Fig 4e for node 14, Fig 4f for node 16). Taking black node 9 in Fig 4c as an example, black node 9 is compared to each of its white children (node 8, 13). 8 is smaller than 9, so change node 8 and its descendants (node 5, 6, 7) to grey. 13 is larger than 9, so change node 13 to black and node 9 to grey.
3. 3 and 4 are smaller than 10, so change node 3 and 4 to grey. Output node 10 and 16 as L-scope candidate boundaries, as shown in Fig 4g.

### The Composite Kernel for Hedge Scope Detection

The hedge scope detection task falls within the scope of semantic analysis of sentences exploiting syntactic structure. To integrate lexical and syntactic information, the composite kernel consisting of the polynomial kernel and the convolution tree kernel is applied to our system.

#### The polynomial kernel

**Fig 5:**
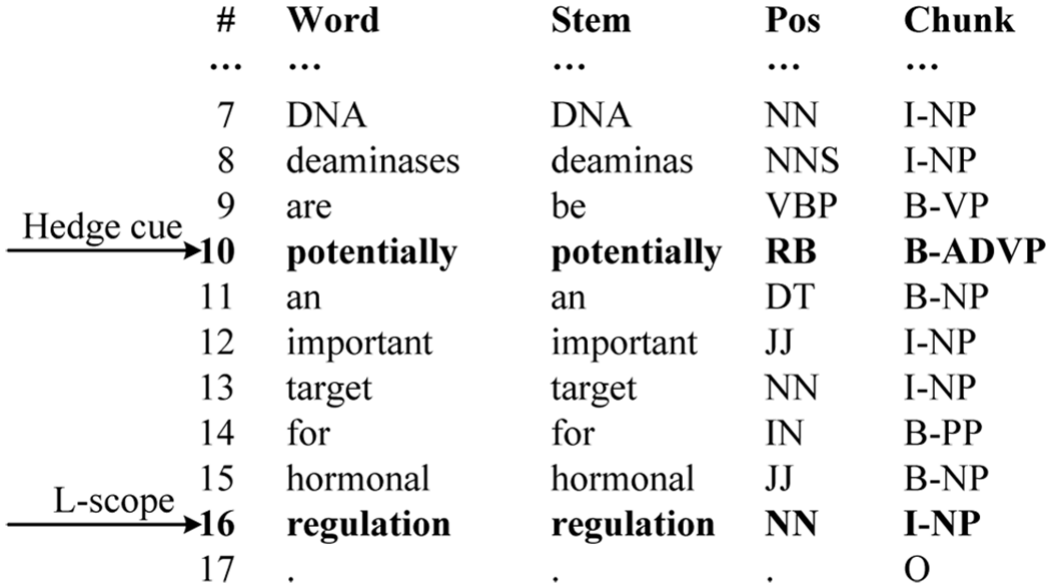
The partial lexical information of sentence 1 preprocessed by GENIA Tagger.

The polynomial kernel *K*_*poly*_(*x*_*i*_, *x*_*j*_) = (*x*_*i*_ ⋅ *x*_*j*_+1)^*d*^ is applied to obtain lexical information. The *d*-th polynomial kernel function can build an optimal separating hyperplane which takes into account all combination of features up to *d*. The parameter *d* is set to 2 in this paper. The **lexical features** used in the polynomial kernel are listed as follows:

- *CandidateToken*(*i*)(*i* = −3, −2, −1,0, 1, 2, 3)
- *CandidateStem*(*i*)(*i* = −3, −2, −1, 0, 1, 2, 3)
- *CandidatePos*(*i*)(*i* = −3, −2, −1, 0, 1, 2, 3)
- *CandidateChunk*(*i*)(*i* = −3, −2, −1, 0, 1, 2, 3)
- *HedgeToken*(*i*)(*i* = −3, −2, −1, 0, 1, 2, 3)
- *HedgeStem*(*i*)(*i* = −3, −2, −1, 0, 1, 2, 3)
- *HedgePos*(*i*)(*i* = −3, −2, −1, 0, 1, 2, 3)
- *HedgeChunk*(*i*)(*i* = −3, −2, −1, 0, 1, 2, 3)
- *Distance: The number of tokens from the hedge cue to its candidate*

where *i* specifies the relative position from the current token. For example, *CandidateToken*(0) denotes the current candidate token, *CandidateToken*(−1) is the first token to the left, *CandidateToken*(1) is the first token to the right, etc. Using the sentence 1, its partial lexical information preprocessed by GENIA Tagger is shown in Fig 5. And the lexical features of the L-scope candidate “*regulation*” for the cue “*potentially*” are listed in Table 1.

**Table 1:** The lexical features of the L-scope candidate “*regulation*”.

Feature name	*i* = -3	*i* = -2	*i* = -1	*i* = 0	*i* = 1	*i* = 2	*i* = 3
***CandidateToken*** **(i)**	target	for	hormonal	regulation	.	null	null
***CandidateStem*** **(i)**	target	for	hormonal	regulation	.	null	null
***CandidatePos*** **(i)**	NN	IN	JJ	NN	.	null	null
***CandidateChunk*** **(i)**	I-NP	B-PP	B-NP	I-NP	O	null	null
***HedgeToken*** **(i)**	DNA	deaminases	are	potentially	an	important	target
***HedgeStem*** **(i)**	DNA	deaminas	be	potentially	an	important	target
***HedgePos*** **(i)**	NN	NNS	VBP	RB	DT	JJ	NN
***HedgeChunk*** **(i)**	I-NP	I-NP	B-VP	B-ADVP	B-NP	I-NP	I-NP
***Distance***	**6**						

#### The convolution tree kernel

The convolution tree kernel is used to obtain syntactic information, which is defined as follows:

$$\begin{array}{c} K_{tree}\left(T_{1},T_{2}\right)=\sum_{n_{1}\in N_{1},n_{2}\in N_{2}}\Delta \left(n_{1},n_{2}\right) \end{array}$$ (1)

where *N*_1_ and *N*_2_ are the sets of all nodes in trees *T*_1_ and *T*_2_, and Δ(*n*_1_, *n*_2_) evaluates the number of the common sub-trees rooted at *n*_1_ and *n*_2_, which can be computed by the following recursive rules:

1. if the productions at *n*_1_ and *n*_2_ are different, then Δ(*n*_1_, *n*_2_) = 0;
2. else if both *n*_1_ and *n*_2_ are pre-terminals, then Δ(*n*_1_, *n*_2_) = *λ*;
3. else, $\Delta (n_{1},n_{2})=\lambda \prod_{k=1}^{♯ch(n_{1})}(1+\Delta (ch(n_{1},k),ch(n_{2},k)))$,

where ♯*ch*(*n*_1_) is the number of children of *n*_1_ in the tree, *ch*(*n*, *k*) is the *k*-th child node of *n*, and *λ* (0 < *λ* < 1) is the decay factor.

Syntactic structure information in phrase and dependency trees can be captured by the convolution tree kernel. Phrase structure usually provides the nearer syntactic information of cues. Meanwhile, dependency structure can offer the farther syntactic information between cues and scopes [22]. Both phrase and dependency structures are effective for scope detection. We consider the two syntactic structures as features.

**Fig 6:**
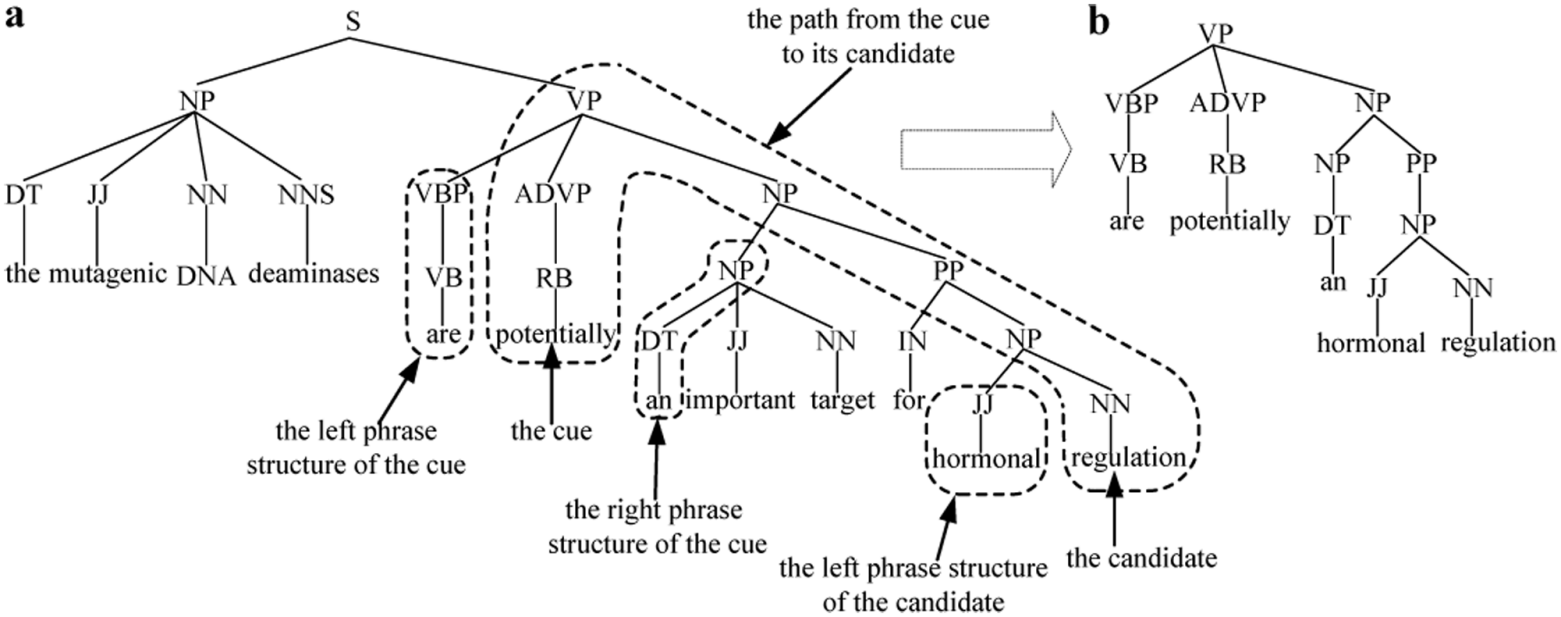
Phrase features extraction. (a) The phrase tree segment of sentence 1; (b) Phrase features.

**Phrase features**: The path from the cue to its candidate boundary, including the phrase structure of the nearest neighbor tokens of both the cue and its candidate boundary, is extracted as phrase features. For the phrase tree segment of sentence 1 in Fig 6a, the phrase features from the cue “*potentially*” to its L-scope candidate “*regulation*” are shown in Fig 6b.

**Fig 7:**
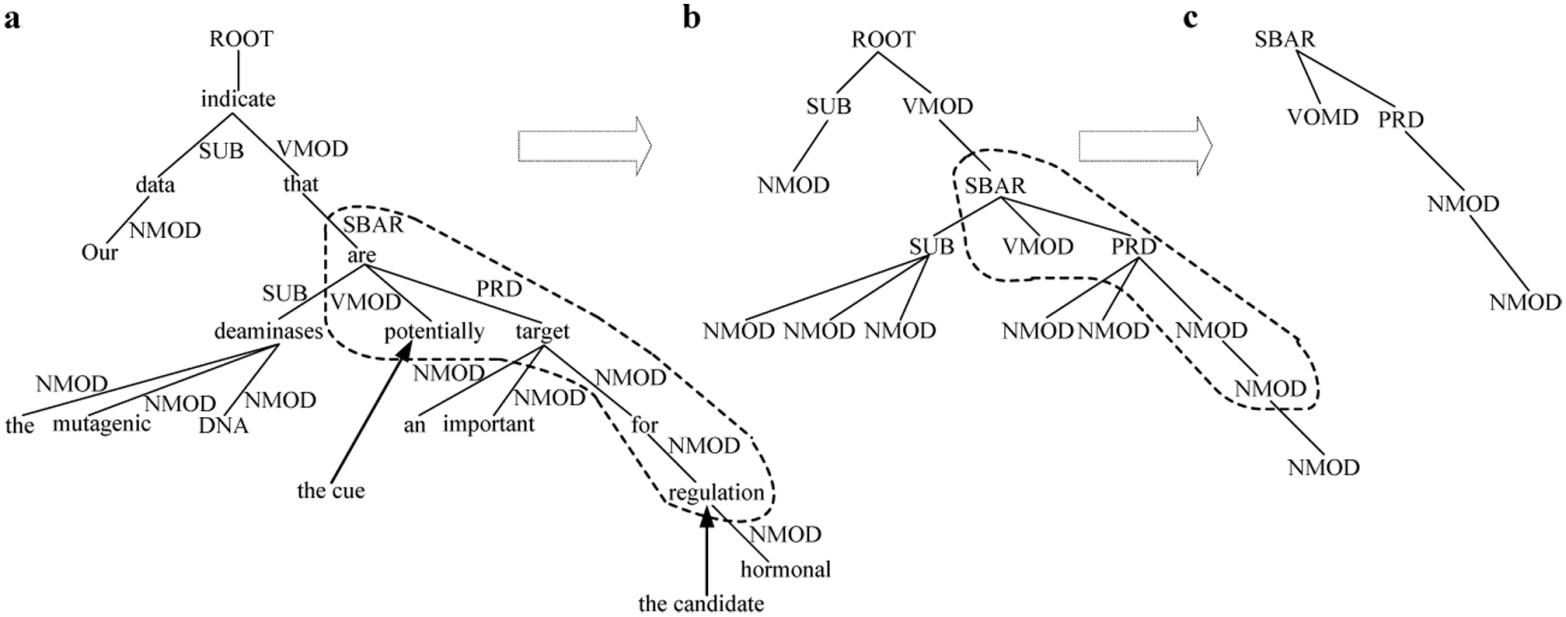
Dependency features extraction. (a) The dependency tree of sentence 1; (b) The dependency node tree; (c) Dependency features.

**Dependency features**: For the dependency tree of sentence 1 as shown in Fig 7a, tree kernel can not represent dependency relation on the arcs (e.g., “*SUB*” relation between node “*indicate*” and node “*data*”). To capture dependency relation, the dependency relation labels are usually used to replace the corresponding tokens on the nodes of original dependency tree as shown in Fig 7b. We define dependency features as critical path from the cue “*potentially*” to its candidate boundary “*regulation*” as shown in Fig 7c.

#### The composite kernel

To integrate the lexical and syntactic features, the composite kernel is defined by combining the above two individual kernels:

$$\begin{array}{c} K_{com}=\gamma K_{tree}+\left(1-\gamma \right)K_{poly} \end{array}$$ (2)

where *γ* (0 ≤ *γ* ≤ 1) is the composite factor. The two syntactic features are combined with the lexical features by the composite kernel respectively. We refer to the combination of the phrase and lexical features as the **phrase_lexical** feature set, the combination of the dependency and lexical features as the **dep_lexical** feature set. The combination of the phrase, dependency and lexical features is also implemented by the composite kernel function in Eq (3), which is called the **phrase_dep_lexical** feature set.

$$\begin{array}{c} K_{com}=\frac{1}{2}\gamma \left(K_{phrase}+K_{dep}\right)+\left(1-\gamma \right)K_{poly} \end{array}$$ (3)

### Postprocessing

Hedge scope is a sequence of tokens including the hedge cue in a sentence. However, sometimes classifiers only predict F-scope or L-scope in a sentence. To guarantee that all scopes are continuous sequences of tokens, we apply the following postprocessing rules to the output of the classifiers.

1. If one token has been predicted as F-scope and one as L-scope, the sequence will start at the token predicted as F-scope, and end at the token predicted as L-scope.
2. If one token has been predicted as F-scope and more than one has been predicted as L-scope, the sequence will start at the token predicted as F-scope and end at the last token predicted as L-scope.
3. If one token has been predicted as L-scope and more than one has been predicted as F-scope, the sequence will start at the first token predicted as F-scope and end at the token predicted as L-scope.
4. If one token has been predicted as F-scope and none has been predicted as L-scope, the sequence will start at the token predicted as F-scope and end at the last token of a sentence.
5. If one token has been predicted as L-scope and none has been predicted as F-scope, the sequence will start at the hedge cue and end at the token predicted as L-scope.
6. If the hedge is passive voice, the scope will start at the subject of the hedge.
7. If the hedge is “*or*”, the scope will start at the first token of the parallel structure conducted by the “*or*” and end at the last token of the parallel structure.

## Results

Experiments are conducted on the CoNLL-2010 Shared Task corpus. We detect the linguistic scope with golden standard cues, which provide 3376 sentences for training and 1047 sentences for testing. The evaluation of hedge scope detection is reported by F1-score for two different levels: tag-level and sentence-level. The tag-level evaluates the performance of the F-scope and L-scope classifiers respectively. The sentence-level evaluation corresponds to the exact match of scope boundaries for each cue.

### Statistics of Positives and Negatives Amount

Table 2 reports statistics of the training and testing instances required for DCBS and TTB. As can be seen from Table 2, the number of negatives is over ten times that of positives in TTB. By using DCBS, negatives decreases to about three times the number of positives. The training and testing instances required for DCBS are much less than that for TTB. It also shows that a few positives in the testing data are dropped due to parsing errors (51 of 1047 F-scope positives and 52 of 1047 L-scope positives are dropped).

**Table 2:** Statistical information of positives and negatives amount.

Method	TTB			DCBS		
**Candidate boundary**	**#Positives**	**#Negatives**	**Ratio**	**#Positives**	**#Negatives**	**Ratio**
**F-scope for training**	3376	41416	1:12	3376	8898	1:2.6
**L-scope for training**	3376	60962	1:18	3376	8001	1:2.4
**F-scope for testing**	1047	14942	1:14	996 (51 dropped)	3133	1:3.1
**L-scope for testing**	1047	18961	1:18	995 (52 dropped)	2779	1:2.8

**Fig 8:**
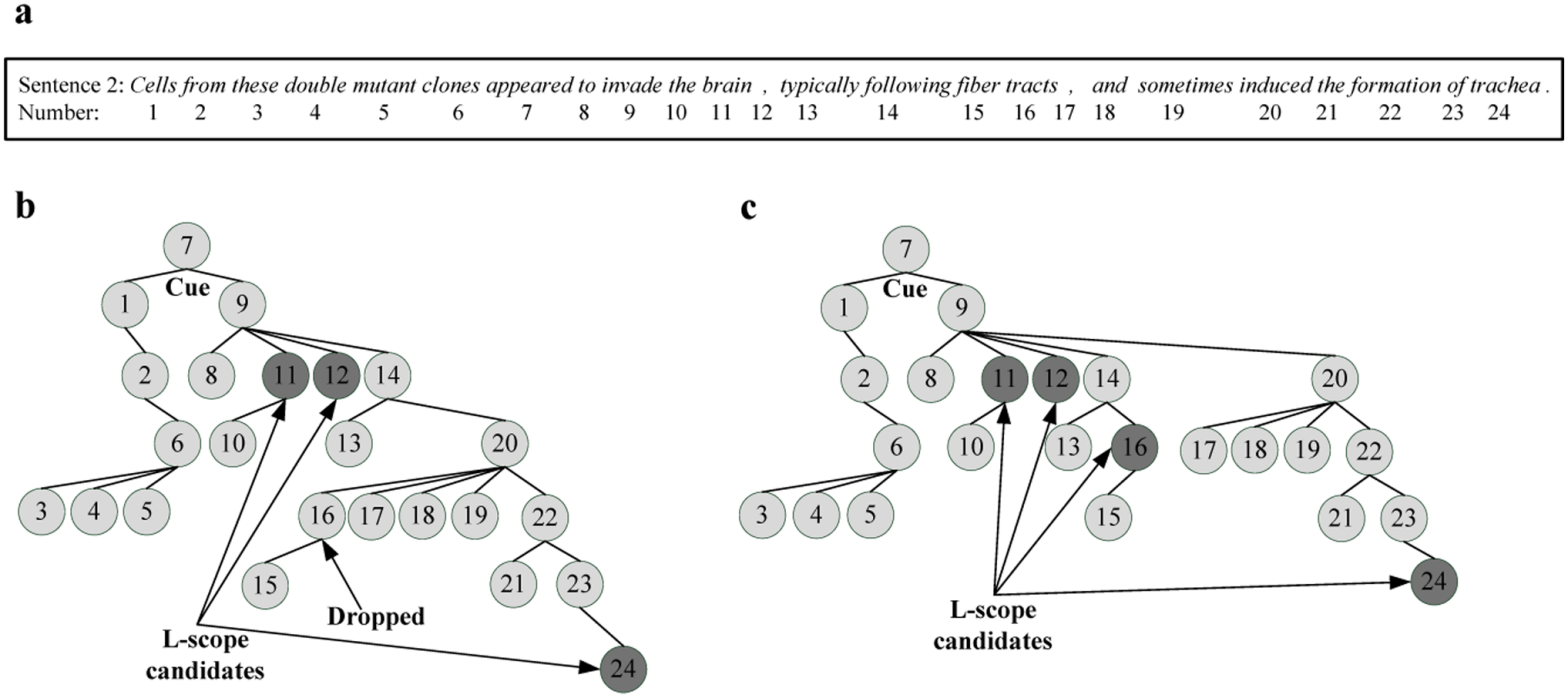
An example of a sentence with the L-scope token dropped due to parsing errors. (a) The sequence number of tokens in sentence 2; (b) DCBS drops the L-scope node 16 due to incorrect dependency tree; (c) DCBS selects the L-scope node 16 accurately based on correct dependency tree.

Fig 8 shows an example of a sentence with the L-scope token dropped due to parsing errors. For the following example sentence 2 in the dataset, Fig 8b shows its dependency tree parsed by Gdep Parser, in which the L-scope token (node 16) is parsed incorrectly. The parsing errors lead to the dropping of a positive instance (the L-scope token “*tracts*”) by DCBS as shown in Fig 8b. The corresponding correct dependency tree for the sentence 2 is shown in Fig 8c, in which the L-scope token (node 16) is selected accurately by DCBS.

Sentence 2: *< xscope > Cells from these double mutant clones < cue > appeared < /cue > to invade the brain, typically following fiber tracts < /xscope >, and sometimes induced the formation of trachea*.

The dropped positives (51 F-scope positives and 52 L-scope positives) account for 4.92% of the total number of existing positives. However, the subsequent experiments show that DCBS could offset the loss of the dropped positives.

### Hedge Scope Detection Performance

#### Effects of candidate boundary selection

**Fig 9:**
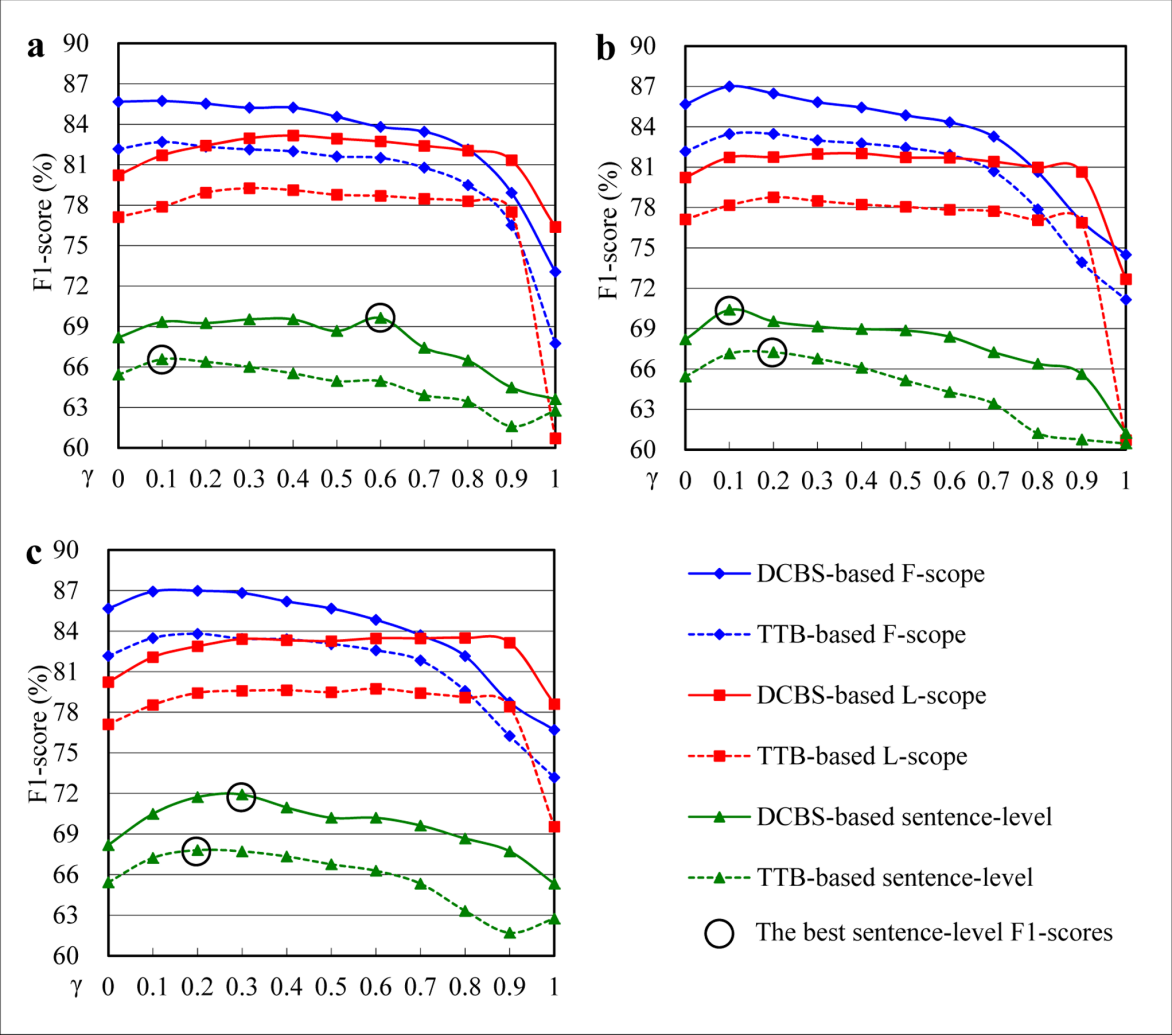
DCBS vs. TTB with the different composite factor *γ*. (a) The phrase_lexical feature set (*p* = 8.05e-04 for sentence-level F1-scores); (b) The dep_lexical feature set (*p* = 6.92e-03 for sentence-level F1-scores); (c) The phrase_dep_lexical feature set (*p* = 2.06e-04 for sentence-level F1-scores). All *p*-values < 0.01 for sentence-level F1-scores.

We compare DCBS with TTB on the tag-level and sentence-level F1-scores. Statistical significance analysis between DCBS and TTB is performed by Student *t*-tests using sentence-level F1-scores. Fig 9 shows the F1-score curves with different composite factor *γ*. We vary *γ* from 0 (sole lexical features) to 1 (sole syntactic features) with an interval of 0.1. The phrase_lexical (Fig 9a), dep_lexical (Fig 9b) and phrase_dep_lexical (Fig 9c) feature sets are investigated in our experiment. From Fig 9, we can see that:

1. For the three feature sets, DCBS steadily outperforms TTB in terms of tag-level and sentence-level with *γ* varying from 0 to 1, though a few positives are dropped due to parsing errors.
2. All *p*-values of the three feature sets are less than 0.01 when compared sentence-level F1-scores of DCBS with that of TTB. Statistical analysis shows significant differences between DCBS and TTB.
3. Both the polynomial kernel with lexical features (*γ* = 0) and the tree kernel with syntactic features (*γ* = 1) could get acceptable results. The sole polynomial kernel outperforms the sole tree kernel. The composite kernel combining lexical and syntactic features with appropriate composite factor *γ* could achieve higher F1-score than either one of them.
4. The performance of F-scope classifiers is usually better than that of L-scope classifiers. The main reason is that the distance of L-scope to its cue is longer than that of F-scope in a sentence. The longer the distance from the scope boundary to its cue is, the harder the scope detection is.

The best sentence-level F1-scores of DCBS and TTB with the three feature sets (marked with solid-circle in Fig 9) are summarized in Table 3. Even though the number of the training instances used for DCBS is less than that for TTB and the used feature sets are the same, the best F1-scores are improved using DCBS. Furthermore, the time required for training and testing is significantly reduced. For example, when using the phrase_dep_lexical feature set, DCBS achieves the best F1-score 71.92% under the condition *γ* = 0.3, which is 4.11% higher than that of TTB under the condition *γ* = 0.2. Meanwhile, the time of training and testing is reduced (19.81min. → 2.17min., 3.41min. → 0.47min.) by applying DCBS.

**Table 3:** Performance comparison of the best DCBS and TTB systems with the different feature sets.

Feature set	Phrase_lexical		Dep_lexical		Phrase_dep_lexical	
**Method**	**TTB**	**DCBS**	**TTB**	**DCBS**	**TTB**	**DCBS**
**F1-score(%)**	66.57(*γ* = 0.1)	69.63(*γ* = 0.6)	67.24(*γ* = 0.2)	70.39(*γ* = 0.1)	67.81(*γ* = 0.2)	71.92(*γ* = 0.3)
**Training time**	18.92min.	2.43min.	7.59min.	1.23min.	19.81min.	2.17min.
**Testing time**	3.08min.	0.46min.	1.16min.	0.19min.	3.41min.	0.47min.

#### Effects of syntactic features

**Fig 10:**
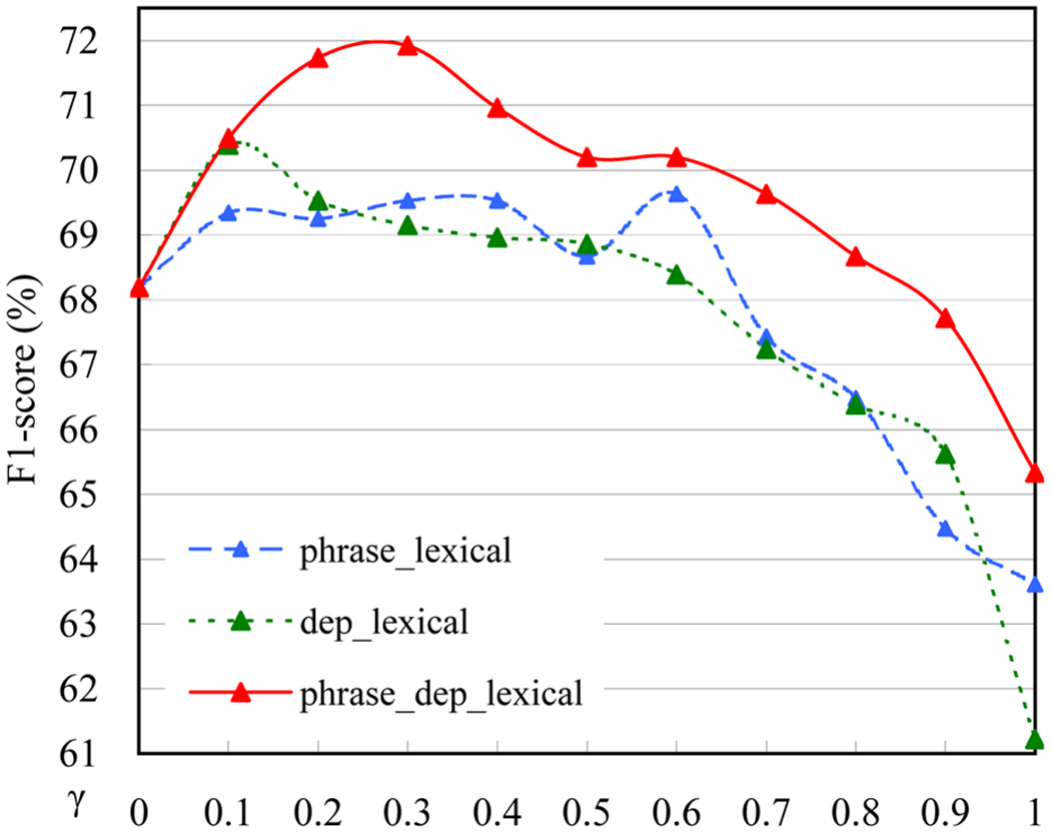
Effects of different syntactic features in DCBS. Student *t*-test for phrase_dep_lexical vs. phrase_lexical results in a *p*-value of 2.14e-02; student *t*-test for phrase_dep_lexical vs. dep_lexical results in a *p*-value of 1.44e-02 (all *p*-values < 0.05).

To obtain the better effects of the syntactic features, the F1-score curves of sentence-level with the three feature sets depicted in Fig 9 are extracted and shown in Fig 10. From Fig 10 we can see that the trends of the three curves are similar. All starts from the initial F1-score of the sole lexical features (68.19%), and then increases to the individual highest F1-score, finally falls below the initial F1-score. Generally, the results with the syntactic features are better than those without them. Both the phrase and dependency features are effective in hedge scope detection and the combination of the two syntactic features outperforms either one of them obviously. The addition of the two syntactic features can improve F1-score from 68.19% to 71.92% (3.73% increases). *P*-values indicate that adding dependency and phrase structured features is statistically significant.

Table 4 compares the performance of the structured syntactic features with the flat syntactic features. Compared with the sole lexical features (68.19%), flat syntactic features improve the performance slightly. The structured phrase and dependency features outperform the corresponding flat features by 1.44% and 2.10% in F1-score, respectively. Moreover, the combination of the two structured syntactic features significantly improves F1-score by 3.53% (from 68.39% to 71.92%). *P*-values by a 10-fold cross-validation on the training data set clearly show significant differences between structured syntactic features and flat syntactic features (all *p*-values < 0.01).

**Table 4:** Structured syntactic features vs. flat syntactic features.

Feature set	F1-score with flat features	F1-score with structured features	*p*-values
**Phrase_lexical**	68.19%	69.63% (1.44 ↑)	4.45e-03
**Dep_lexical**	68.29%	70.39% (2.10 ↑)	9.02e-09
**Phrase_dep_lexical**	68.39%	71.92% (3.53 ↑)	2.80e-12

### Summary of the Classification Results of DCBS and TTB

**Fig 11:**
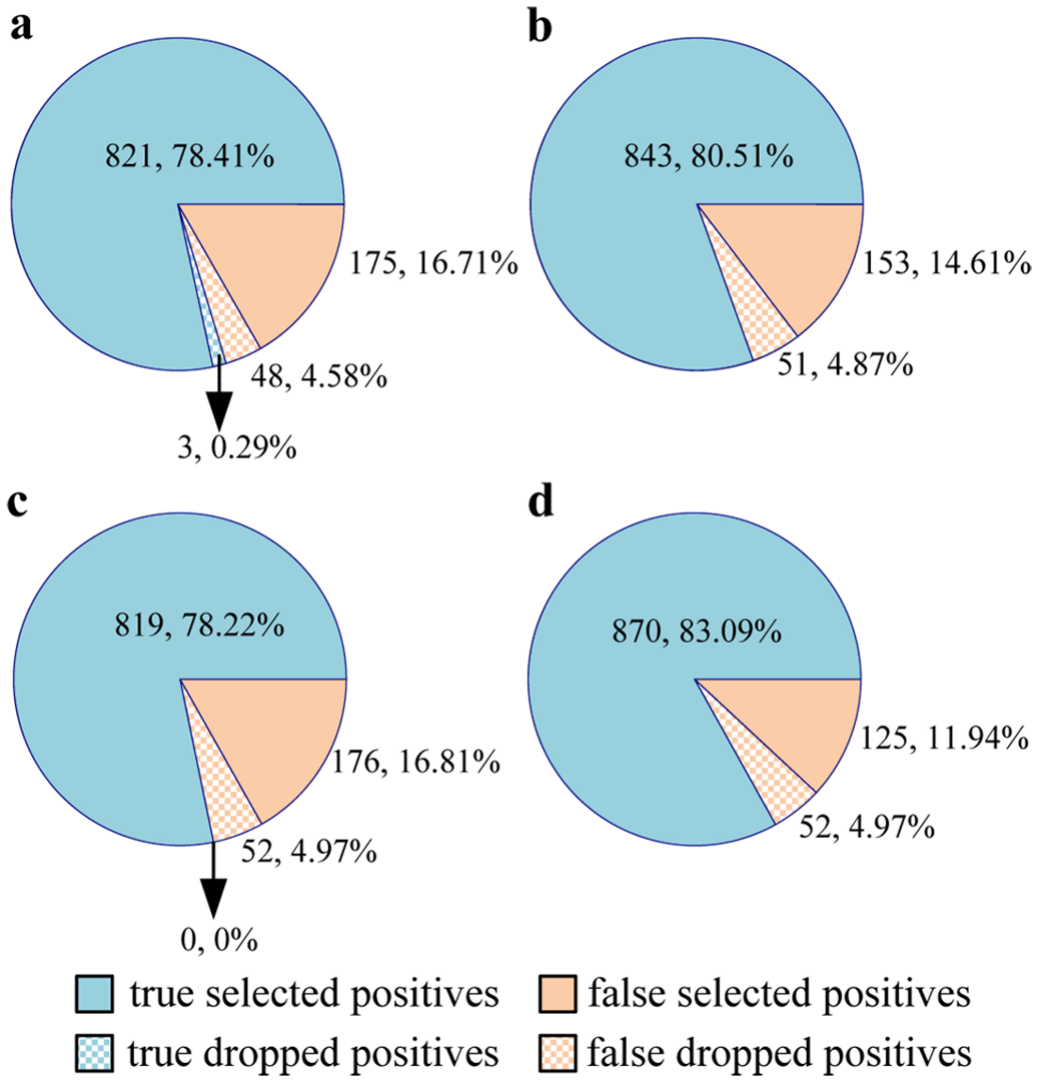
Summary of the tag-level classification results of DCBS and TTB. (a) F-scope classification results with TTB; (b) F-scope classification results with DCBS; (c) L-scope classification results with TTB; (d) L-scope classification results with DCBS.

The detailed F-scope and L-scope classification results of DCBS (*γ* = 0.3) and TTB (*γ* = 0.2) with the phrase_dep_lexical feature set are summarized in Fig 11. The 1047 positives in testing data are divided into two categories: **selected positives** and **dropped positives** by DCBS. For 51 dropped positives in F-scope classification, the true positives classified by TTB are only three as shown in Fig 11a. Meanwhile for 52 dropped positives in L-scope classification, no positive is correctly classified by TTB as shown in Fig 11c. On the other hand, for the selected positives, DCBS-based F-scope classifier detects 22 true positives (Fig 11a and 11b) and DCBS-based L-scope classifier identifies 51 true positives (Fig 11c and 11d) more than TTB-based classifiers. These results indicate that DCBS could offset the loss of the dropped positives.

### Comparison with Related Work

Table 5 compares the results of our method with the state-of-the-art results on golden standard cues of the CoNLL-2010 Shared Task corpus. From Table 4 we can see that our method achieves the best performance with an F1-score of 71.92%. Zhou et al. [9] and Velldal et al. [12] also employed dependency and phrase parsing trees. Zhou et al. [9] employed decision tree to construct the dependency constraint set and phrase constraint set based on dependency structure and phrase structure respectively, which were used to generate the syntactic constraint features for hedge scope detection. They reached 70.28% F1-score. Velldal et al. [12] combined a rule-based approach over dependency structure and a data-driven approach over phrase structure. They obtained an F1-score of 69.60%. Our method simply combines the structured dependency and phrase features, and outperforms Zhou et al. [9] and Velldal et al. [12] by 1.64% and 2.32% in F1-score, respectively. The performance improvement is obvious. The superiority of our system benefits from our candidate boundary selection method and the structured syntactic representation method.

**Table 5:** Comparison with the related work.

System	Our system	Zhou et al. [9]	Velldal et al. [12]
**F1-score (%)**	71.92	70.28	69.60

## Discussion

We present a dependency-based candidate boundary selection method for hedge scope detection. Experimental results show that our method outperforms most of the state-of-the-art systems. The analysis is as follows.

### Effectiveness of candidate boundary selection

The proposed DCBS is effective for hedge scope detection. The absolute superiority of DCBS for scope detection benefits from the removal of a large number of confusing negatives generated by TTB. From the above experimental results, we can conclude that DCBS has the following advantages over TTB.

1. Simple and efficient: DCBS is simple, which selects candidates only based on dependency tree. Moreover, the method could reduce the training and testing cost significantly by decreasing the number of candidates.
2. Balance instance bias: TTB generates a large number of negatives and results in instance bias for classifiers. DCBS can filter out the most likely non-boundary tokens of a given cue and mitigate potential imbalance of positives and negatives.
3. Enhance the discriminability of instances: TTB takes the boundary tokens as positives and the other tokens including the neighbors of boundary tokens as negatives. As adjacent tokens have similar structure and contextual information, the boundaries and their neighbors are extremely difficult to distinguish for classifiers. DCBS can eliminate most of the neighbors of boundaries to improve the classification performance.

### Effectiveness of syntactic features

The composite kernel consisting of the polynomial kernel and the tree kernel is employed to integrate lexical and syntactic information. Lexical features contain semantic and contextual information. Syntactic features capture the structured information. Therefore, syntactic features and lexical features are complementary for hedge scope detection, and their combination could improve the performance further.

Phrase syntactic features represent constituent information of neighbor words, and it is well suitable to capturing the local syntactic information. Dependency syntactic features are compact and can capture global syntactic information between cues and scopes. Thus, the phrase and dependency syntactic features are complementary for hedge scope detection. The combination of the two features could obviously outperform either one of them.

In addition, the structured syntactic features with the tree kernel outperform the flat syntactic features with the polynomial kernel. The main reason is that the polynomial kernel cannot capture the structured syntactic features, while the tree kernel can effectively capture the structured features by counting the number of the common subtrees.

## Conclusions

We present a dependency-based candidate boundary selection method for hedge scope detection which achieves 71.92% F1-score on the CoNLL-2010 Shared Task corpus. Our method is designed along with the heuristics that two tokens with dependency relation should be both in the scope and selects the further token away from the cue as candidate boundary. DCBS can eliminate most of confusing negatives generated by TTB and therefore enhances the discriminability of candidate instances. Our method outperforms previous state-of-the-art methods with respect to performance and efficiency. In addition, the composite kernel consisting of the polynomial kernel and the tree kernel is employed to integrate lexical and syntactic information. The structured phrase and dependency features both outperform the corresponding flat features and the combination of structured phrase and dependency features achieves even better results. To the best of our knowledge, our method obtains the best published results so far on the CoNLL-2010 Shared Task corpus for scope detection.

Besides lexical and syntactic information, semantic information of words plays an important role in hedge scope detection. How to extract the semantic knowledge of words, especially how to calculate the semantic similarity between two cues and combine semantic information with our lexical and syntactic information will be studied in our future work.
